# Effect of Laser Power on Weld Formability and Residual Stress of Unequal Thickness 410 Ferritic Stainless Steel/RCL540 Low-Carbon Alloy Steel

**DOI:** 10.3390/ma17225537

**Published:** 2024-11-13

**Authors:** Yubo Wang, Wei Liu, Wenbiao Gong, Yao Wang, Peng Lü

**Affiliations:** 1 Key Laboratory of Advanced Structural Materials, Ministry of Education and School of Materials Science and Engineering, Changchun University of Technology, Changchun 130012, China; 2 Jilin Province TaiHao Railway Vehicles Facilities Co., Ltd., Changchun 130500, China

**Keywords:** laser welding, weld formability, mechanical properties, residual stress

## Abstract

In this paper, the butt joint of unequal thickness 410 ferritic stainless steel and RCL540 low-carbon alloy steel sheets are realized by laser welding. The effects of different laser powers on weld formability, mechanical properties, and residual stress in the welding process are investigated. It is observed that with increasing laser power, the heat accumulates at the bottom of the molten pool and weld metal, causing the ratios of upper and lower melt widths to decrease. The tensile test results show that all specimens fractured in the weak zone of the base metal on the stainless steel side at 10 mm from the weld seam. The residual stress distributions of the specimens are calculated using ABAQUS 2022 software and compared with the measurements of the blind-hole method. It is found that the stainless steel side produces tensile stresses, with the power increase offset by compressive stresses in the base metal. When the laser power is 1200 W, the welded joint has the best weld formability and mechanical properties and the least residual stress. The upper and lower melt width ratio is 1.17, the maximum microhardness of the weld metal is 374.7 HV, the maximum test force and tensile strength are 5617.5 N and 468.12 MPa, respectively, and the minimum values of the transverse and longitudinal stresses are −45.8 MPa and −106.4 MPa, respectively.

## 1. Introduction

Ferritic stainless steel–low-carbon steel-welded structures combine the high strength and toughness of carbon steel and the corrosion resistance of ferritic stainless steel. This welded structure of dissimilar steels meets the different material requirements of the industry. Nevertheless, stainless steel–low-carbon steel-welded has some problems in industry. Because of the excessive welding energy and welding temperature, the conventional welding process makes the welded joint prone to cracking during the process. Moreover, the heat-affected zone of welded joints has a strong tendency to cold cracking caused by coarse grain embrittlement, which limits the engineering applications of stainless steel–low-carbon steel composite parts, as identified by Samek et al. (2020), Efthymiadis et al. (2018), and Gu et al. (2023) [1,2,3].

Weld quality is affected by the size and location of the weld nugget because unequal thickness sheets in comparison to equal thickness sheets can lead to issues like inadequate welding depth of fusion or incomplete penetration of the welded joint. Sun et al. (2023) [4] studied the effect of different butt gaps and welding methods on the microstructure and properties of welded joints of unequal thickness steel plates. With the increased of butt gaps, the hardness of the weld metal decreased, and the tensile strength of welded joints first increased and then decreased. Azzouzi et al. (2019) [5] created spot welding of equal and unequal sheet thicknesses. The results illustrated that in contrast to the nugget diameter growth, which was mostly driven by the weld current, the weld nugget thickness growth was significantly more impacted by the weld time than the weld current. Dong et al. (2019) [6] studied the forming quality and microhardness of welded joints of unequal thicknesses of dissimilar steels at different laser powers. The increase in laser power has little effect on the tensile strength of the joints compared to the base material, while the original fracture location of the joint changes from the base material to the lower hardness heat-affected zone.

To prevent metals from cracking, some researchers have tried to use friction welding (FW), namely, Ratković et al. (2018) and Khidhir et al. (2019) [7,8], and friction stir welding (FSW), namely, Matsushita et al. (2018) and Pankaj et al. (2021) [9,10], to join dissimilar steels. It is found that joining steels of dissimilar chemical, mechanical, thermal, and electrical properties creates many effects. These effects can cause problems in the joining process itself and cause residual stresses to dissimilar steels’ friction welding in the weld seam. FSW is mostly used on light metals, like aluminum and magnesium alloys, per Torabi et al. (2023) [11]. The cost of high-strength metals, such as steels, is more because they require more expensive tools and more sophisticated instrumentation. Some researchers have attempted to weld dissimilar steels using welding techniques such as tungsten inert gas welding (TIGW), specifically, Singh et al. (2019) and Zhang et al. (2019) [12,13], and melt inert gas welding (MIGW), specifically, Cui et al. (2018) and Roopa et al. (2021) [14,15]. However, the conventional TIGW can increase the weld width and reduce the penetration. There are defects such as porosity and inclusion in the weld seam, and the addition of active agent affects the tensile mechanical properties and corrosion resistance of the welded joint. After MIGW, softening of the joint occurs, resulting in a significant decrease in microhardness. As a concentrated heat source, laser welding uses a laser to produce deep and narrow weld seams, and the high-power density of the laser enables rapid heating and cooling Mao et al. (2021) [16]. Therefore, there are many applications for the engineering study on laser welding of dissimilar steels.

In this study, we investigated the weld formability of weld seams with different laser powers. The disadvantage of welding sheets of unequal thickness, which is easily incompletely welded, is solved by comparing the upper and lower melting widths in the case of increasing heat input. We used microhardness and tensile tests to find the weak zone of the specimen to solve the disadvantages of dissimilar steel welding due to weld cracks, resulting in poor mechanical properties. The residual stresses of the specimens are studied by using the blind-hole method and the ABAQUS finite element analysis (FEA), which provides a certain reference for the study of residual stresses in laser welding of dissimilar sheets of unequal thickness.

## 2. Materials and Methods

The experimental metal materials are 160 mm × 80 mm × 1 mm 410 ferritic stainless steel sheets and 160 mm × 80 mm × 2 mm RCL540 low-carbon alloy steel sheets, composed of 410 stainless steel and RCL540 carbon steel, as shown in Table 1.

**Figure 1:**
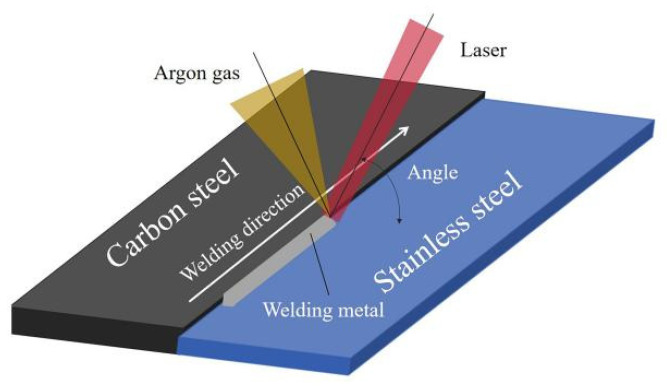
Welding assembly drawing.

The steel surface is ground before welding to remove the impurities on the steel surface, and the steel surface is cleaned with alcohol. Welding was performed with the YLS-2000 laser welder (Anshan Yuchen Technology Co., Ltd., Anshan, China), and the welding assembly drawing is shown in Figure 1. The laser process parameters are as follows: focal length of 300 mm; spot diameter of 0.3 mm; defocusing distance of −1 mm; welding speed of 25 mm·s^−1^; laser beam incidence angle of 70°; and argon gas flow of 20 L·min^−1^. Through a large number of pre-testing experiments, we measured the appropriate laser power range of 900 W to 1400 W, taking three groups in the middle range of the laser power for the experiment; the laser powers are set to be 1200 W, 1100 W, and 1000 W under these experimental conditions.

**Figure 2:**
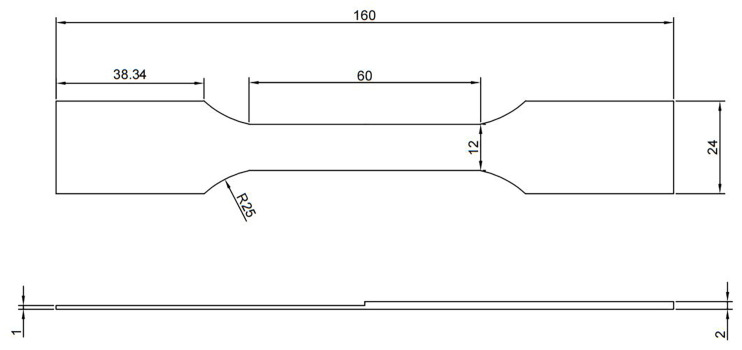
Schematic diagram of tensile specimen.

**Figure 3:**
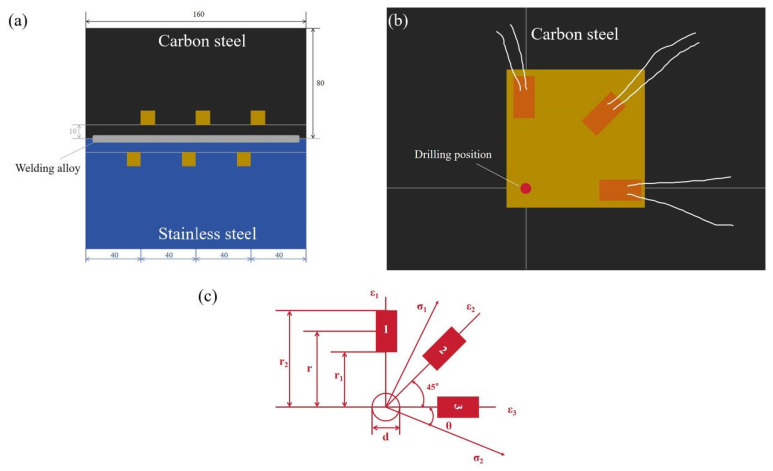
(**a**) Diagram of strain gauge distribution. (**b**) Strain gauge placement and drilling position. (**c**) Schematic diagram of strain rosette.

After the welding process, metallographic specimens measuring 20 mm × 10 mm are machined perpendicular to the welding direction. Subsequently, the joints undergo polishing and are then etched for a duration of 10 s using a solution consisting of 4% HNO_3_ alcohol and FeCl_3_ (FeCl_3_:HCl:H_2_O = 2:1:10). The optical morphology of the joints are investigated using a Leica DMI8 optical microscope (Leica Microsystems (Shanghai) Trading Co., Ltd., Shanghai, China). The microhardness of the joints are measured using a FM700 microhardness tester (Xi’an Minsks Testing Equipment Co., Ltd., Xi’an, China) with an indentation load of 100 gf employed for each test and the dwell time of 15 s, and the distance between each two points is 0.25 mm. The tensile test of the joints is conducted using a WDW-200 material testing machine (Jilin Guanteng Automation Technology Co., Ltd., Changchun, China) at room temperature and a constant strain rate of 1 mm·min^−1^, and the schematic diagram of the tensile specimen is shown in Figure 2. Each process parameter is analyzed by averaging 3 sets of specimens. The residual stresses are determined using the JHYC-20 static strain gauge test system (Nanjing Juhang Technology Co., Ltd., Nanjing, China). Strain rosettes are affixed with 502 glue and left to stabilize for a duration of 2 days, after which the blind-hole method is employed to measure the residual stresses in the weldments. In this experiment, the depth of the drill hole is 1 mm, and the diameter of the drill solenoid is 1.5 mm. A diagram of the strain gauge distribution, strain gauge placement, and drilling position are shown in Figure 3a,b.

The blind-hole method is based on the principle of alleviating residual stresses within a metal by drilling a hole. Initially, a strain rosette is affixed onto the metal surface. According to the blind-hole method, a hole is drilled at a specific diameter and depth in a location within the member where residual stresses exist, and the metal removal within the hole results in the release of the residual stresses. The presence of a blind hole will induce a new residual stress field due to the release of strain. Throughout the drilling process, the strain rosette surrounding the hole will track the released strain and calculate the initial residual stresses at the test point using the corresponding formula [17]. According to Kirsch’s equation for the through-hole method, the relationships between residual stress and released strain by strain gauge are as follows [18,19]:

(1) $$\sigma_{1,2}=\frac{\varepsilon_{1}+\varepsilon_{3}}{4A}\pm \frac{\sqrt{{\left(\varepsilon_{1}-\varepsilon_{3}\right)}^{2}+{\left[2\varepsilon_{2}-\left(\varepsilon_{1}+\varepsilon_{3}\right)\right]}^{2}}}{4B}$$

(2) $$tan\;2\theta =\frac{\varepsilon_{1}+\varepsilon_{3}-2\varepsilon_{2}}{\varepsilon_{3}-\varepsilon_{1}}$$

(3) $$A=\frac{\varepsilon_{1}+\varepsilon_{3}}{{2\sigma }_{1}}$$

(4) $$B=\frac{\varepsilon_{1}-\varepsilon_{3}}{2\sigma_{1}}$$

In Figure 3c, ε_1_, ε_2_, and ε_3_ are the release strains of corresponding directions after drilling; σ_1_ and σ_2_ are the maximum and minimum principal stresses; θ is the principal stress’s direction angle; A and B are strain release coefficients, which are related to material properties; Young’s modulus and Poisson’s ratio are obtained by tensile test with JMatPro 7.0 simulation, namely, E = 211 GPa and ν = 0.29, respectively. In the formula, A and B are constant in the elastic range. The plastic strain varies with the stress level when the metal at the edge of the hole yields, and the strain release coefficients change as a result. The parameter values of stress release coefficients after calculation are A = −0.096 and B = −0.286.

During laser welding, free expansion and compression of the specimens are impeded due to influence from external loads and constraints to compensate for the expansion and compression due to the temperature change of the laser energy. As the WM cools, the following residual stresses exceeding the yield point of the metal are formed in the molten pool and its vicinity: (1) longitudinal stress parallel to the welding direction (σ_x_), and (2) transverse stress perpendicular to the welding direction (σ_y_) [20]. For studying the distribution of residual stresses in 410 ferritic stainless steel and RCL540 low carbon alloy steel, the residual stresses are measured using the blind-hole method. Starting from the welding origin (0 mm), six residual stress measurement points are taken at point 1 (40 mm), point 2 (80 mm), and point 3 (120 mm) on both sides of the weld seam (10 mm), as shown in Figure 3a.

## 3. Results and Discussion

### 3.1. Weld Formability

**Figure 4:**
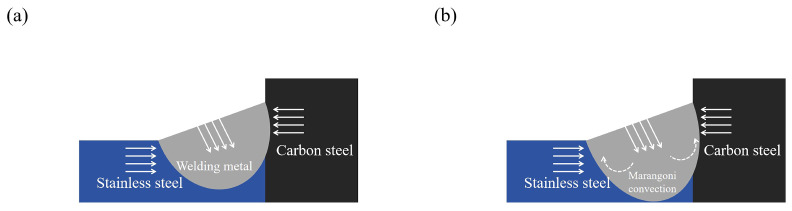
(**a**) Schematic diagram of welding joint forming. (**b**) Marangoni convection.

**Figure 5:**
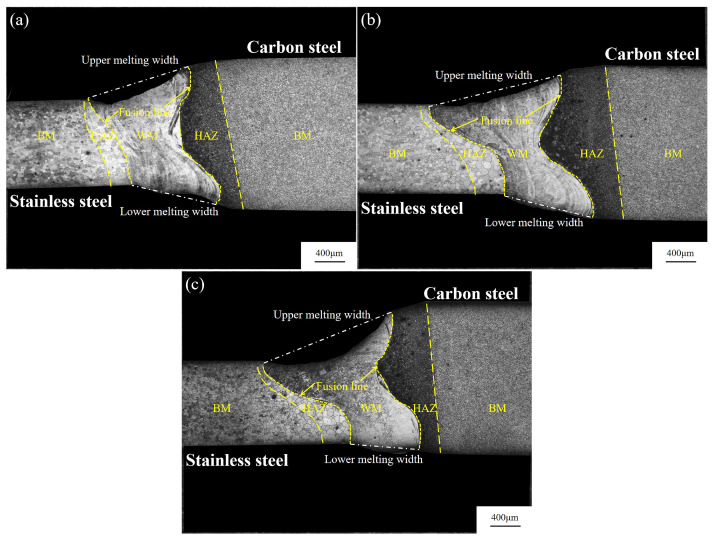
Optical morphology of the joint at (**a**) 1200 W, (**b**) 1100 W, and (**c**) 1000 W.

Laser welding can be categorized according to the following different characteristics of weld formation: (1) When laser power density is less than 10^6^ W·cm^−2^, welding is carried out by heat conduction. The welding process transfers energy from the metal surface to the inner surface through the heat transfer effect, causing the metal inside the molten pool to melt into a liquid metal flow. After welding, the melt width is more than the melt depth. (2) When laser power density is more than 10^6^ W·cm^−2^, welding is carried out by keyhole welding. The welding process uses the evaporation of the metal surface to generate pressure and combines with the surface tension of the liquid metals so that the molten pool surface is concave to the internal metal and forms the keyhole. After welding, the melt width is less than the melt depth [21]. Melting width measurement results, heat input, and power density calculation results at welding speed of 25 mm·s^−1^ are shown in Table 2. The heat input (HI) and power density (P_D_) are calculated by the following formulas:

(5) $$HI=\frac{P}{V}$$

(6) $$P_{D}=\frac{P}{\pi r^{2}}$$

where P is laser power, V is welding speed, and r is laser spot radius. In Figure 4a, the weld metal is subject to three forces as it melts: (1) the rigid binding force between the steel BM, (2) the reaction force caused by the metal melting under the laser beam, and (3) the reaction force caused by the metal evaporating under the laser beam [22]. Temperature has an effect on liquid surface tension and according to the Eötvös’s law, the higher the temperature, the less the surface tension:

(7) $$\sigma =\frac{k_{r}}{V^{\frac{2}{3}}}\left(T_{c}-T\right)$$

where V is molar volume; T is thermodynamic temperature; T_c_ is the liquid-gas critical point temperature; and kr is the temperature coefficient, whose value is roughly the same for all liquid metals (6.4 × 10^−8^ J·K^−1^mol^−2/3^). Carbon steel and stainless steel in the laser heat under the melting and mix to form a metal molten pool whose center temperature is higher than the molten pool edge temperature. The surface tension at the center pool is less than the edge pool, which causes the Marangoni effect [23]. Within the limits of the carbon steel and stainless steel BM, the metal in the middle of the molten pool will flow to the edge as a result of this surface tension gradient, and the liquid metal will return to the top part of the molten pool. This flow model is illustrated in Figure 4b. The morphology of the joints in Figure 5 shows how the turbulent liquid metal in the middle and upper part of molten pool eventually cools and crystallizes to form the WM.

The optical morphology of the joints obtained under different laser powers are shown in Figure 5; the specimens are completely penetrated, and the welded joints are of high quality with no visible defects. The color shades of the weld metal (WM), the heat-affected zone (HAZ), and the base metal (BM) are different, which is mainly due to the differences in the composition of the WM and BM. In addition, there are differences in the welding thermal cycle temperature caused by the two sides of the organization [24]. The laser melting mode can be judged from the weld morphology and laser power density in keyhole mode. As laser power and heat input increase, the upper and lower melting width ratios decrease to 1.81 at 1000 W and 1.43 when the power increases to 1100 W. When the laser power is increased to 1200 W, the upper and lower melting width ratio reaches a minimum of 1.17. Figure 5a–c displays the results due to the laser power density and heat input increase as the laser power increases, resulting in a keyhole deeper into the specimen. The specimen has a higher absorption of the laser, and the energy is concentrated mainly in the lower part of the molten pool.

### 3.2. Mechanical Properties

**Figure 6:**
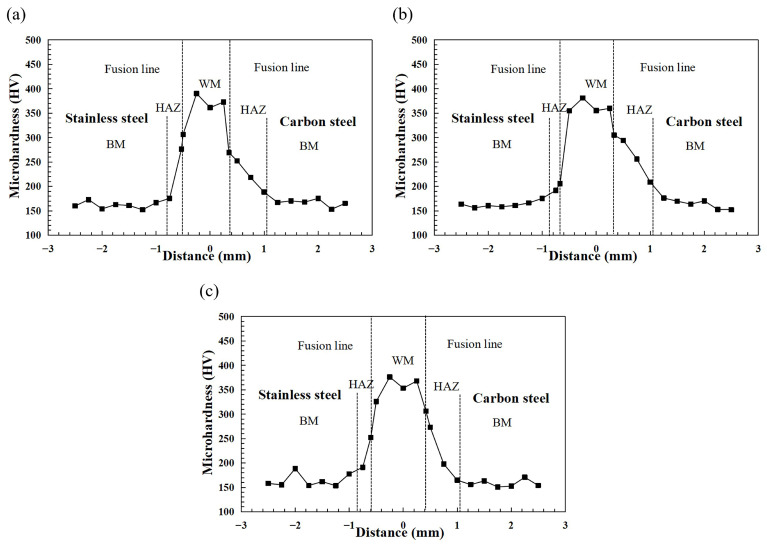
Microhardness of the joints obtained under dissimilar laser power. (**a**) 1200 W, (**b**) 1100 W, and (**c**) 1000 W.

As shown in Figure 6, Vicker’s hardness in the middle of the joints and the hardness of the BM fluctuate more gently, while the WM fluctuates more violently. The average hardness of the stainless steel BM at 1200 W is 161.2 HV, and that of the carbon steel BM is 166.5 HV. The minimum value of 153.1 HV on the carbon steel side occurs in the tempered softened zone. It is obvious from Figure 6a that the hardness increases from the stainless steel and carbon steel BM to WM, which is very obvious in the HAZ. The high hardness of the WM is mainly due to the formation of high-hardness martensite in the columnar crystals of the WM [25]. Remelting of the stainless steel and carbon steel BM form the WM, which has an average hardness of 374.7 HV at 1200 W, 362.8 HV at 1100 W, and 365.7 HV at 1000 W.

**Figure 7:**
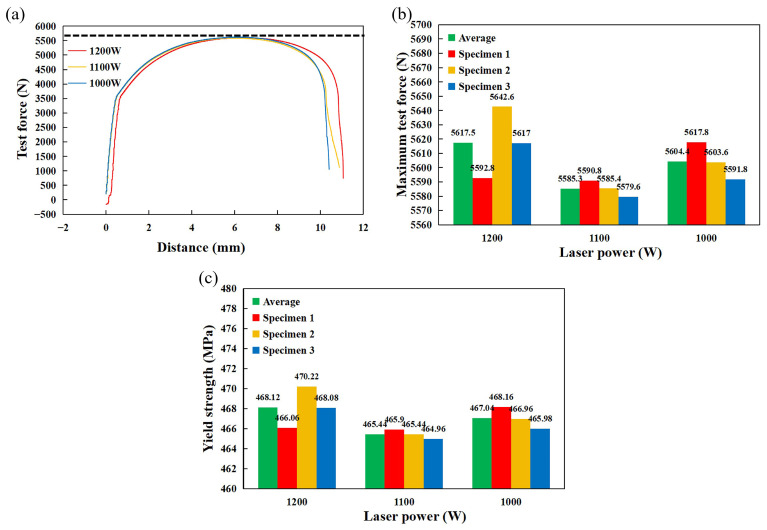
Tensile test of the joints. (**a**) Test force-distance curves. (**b**) Maximum test force. (**c**) Tensile strength.

The tensile curves for each set of laser-welded joint specimens are shown in Figure 7a, and the values and average values of the tensile experiments are shown in Figure 7b,c. The average maximum test force and yield strength are 5617.5 N and 468.12 MPa at 1200 W, respectively, 5585.3 N and 465.44 MPa at 1100 W, respectively, and 5604.4 N and 467.04 MPa at 1000 W, respectively. It is shown that increasing the laser power slightly increases the average hardness and tensile properties of specimens, and laser welding can improve the mechanical properties of specimens. All specimens fracture in the stainless steel BM 10 mm to 20 mm, which is the weak zone of the specimens. Therefore, the specimens will not fracture at the WM due to possible little amounts of welding defects. To explore the effects of residual stresses on the welded joint, the blind-hole method drilling location is selected in the distance from the WM 10 mm position.

**Figure 8:**
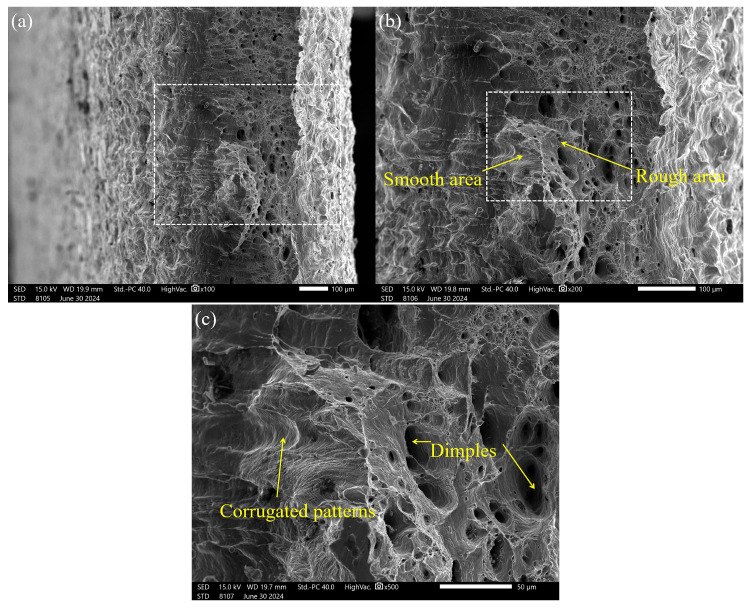
Fracture surface morphology. (**a**) Macro-morphology. (**b**) Fracture area morphology. (**c**) Rough area and smooth area morphology.

Figure 8a shows the middle of the fracture area of the specimen fracture. The macro-morphology fracture has an obvious cupped neck, which is due to the typical toughness fracture. The fracture area that separates the smooth area from the rough area is chosen for observation in Figure 8b. It is evident that there are differences in the fracture height and the fracture area, with rough and smooth areas in the specimen. While the rough area has a lot of flocculent distributions, the smooth area has a regular flake pattern. The fracture area is further magnified for observation in Figure 8c, with the magnifications as follows: (1) Tear dimples of different sizes and depths are found in the rough area of the fracture area, which is a tensile fracture. (2) Obvious arc-shaped corrugated patterns on the striped and plane are found in the rough area of the fracture area, which is a fatigue fracture [26].

### 3.3. Residual Stress

**Figure 9:**
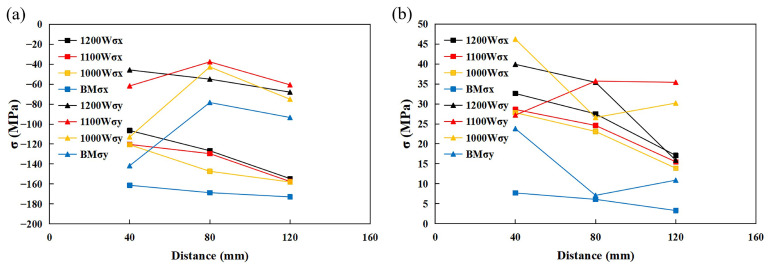
Residual stress measurement results. (**a**) Stainless steel side. (**b**) Carbon steel side.

In Figure 9, the residual stress measurement results with different laser powers are shown. The residual stresses are mainly concentrated on the stainless steel side. The 410 stainless steel side values increased from two to three times more than on the RCL540 carbon steel side. All values are almost negative and show a good uniformity, indicating that most of the stresses applied are compressive stresses, and carbon steel side stresses are mostly tensile stresses [27].

Residual compressive stresses are generated due to plastic deformation in the region affected by thermal stress. The material around it has to resist this deformation, since tensile stresses are generated for the region not affected by thermal stress. Along the welding direction, the compressive stresses increase on the stainless steel side, and the tensile stresses decrease in the carbon steel side. The residual stresses on the stainless steel side decrease with the increase of laser power and are minimized at 1200 W. Although the residual stresses in the carbon steel side are not equal at different powers and positions, they are maximized at 1200 W, presumably due to tensile stresses from the thermal stress offsetting the original compressive stresses within the stainless steel BM. In order to find the reason why the residual stress decreases with increasing laser power, an FEA simulation based on the temperature field is carried out to investigate whether an increase in laser power increases tensile stresses.

### 3.4. Finite Element Analysis

Residual stress analysis requires the interaction of a temperature field and a structural field. The fields’ coupled form is divided into the following two kinds: direct coupled and indirect coupled. (1) Direct coupled considers the temperature field on the structural field, and the structural field effects the temperature field. It is a two-way process, such as friction stir welding. (2) Indirect coupled only considers the temperature field on the structural field, and the structural field hardly effects the temperature field. It is a one-way process, such as laser welding. This suggests that the welding FEA simulation needs lots of processing power and data storage. Thus, so as to ensure the simulation is accurate enough, some assumptions and simplifications must be made [28].

#### 3.4.1. Thermal Analysis

**Figure 10:**
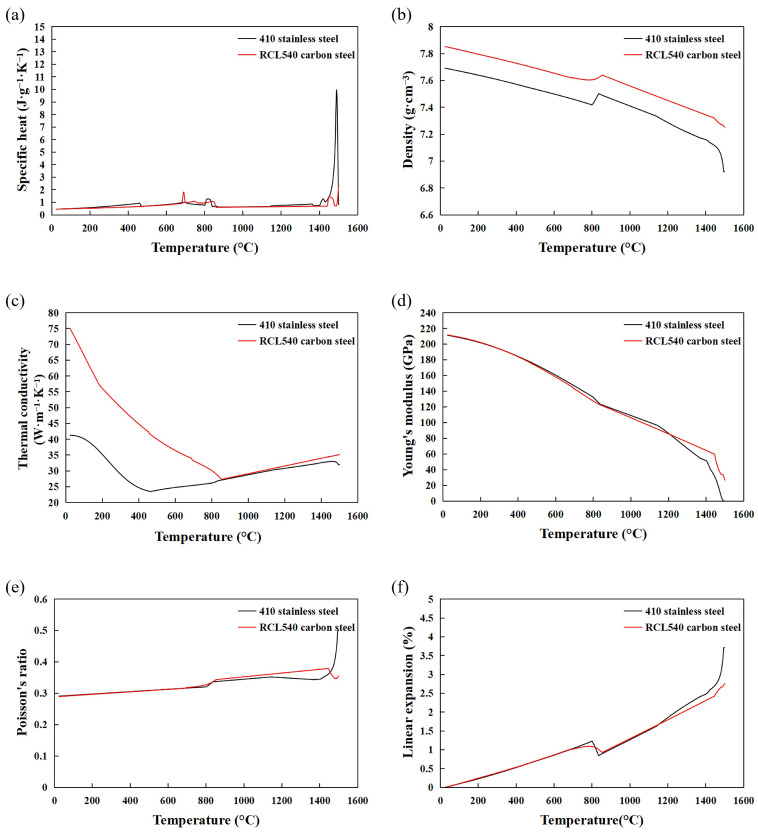
Thermophysical property parameters and thermodynamic performance parameters of 410 ferritic stainless steel and RCL540 low-carbon alloy steel. (**a**) Specific heat. (**b**) Density. (**c**) Thermal conductivity. (**d**) Young’s modulus. (**e**) Poisson’s ratio. (**f**) Linear expansion.

During the welding process, the temperature field is a nonlinear instantaneous thermal analysis problem, with the following differential equation for heat transfer:

(8) $$C\rho \frac{\partial T}{\partial t}=\lambda (\frac{\partial^{2}T}{\partial x^{2}}+\frac{\partial^{2}T}{\partial y^{2}}+\frac{\partial^{2}T}{\partial z^{2}})+\partial Q_{t}$$

where C is specific heat capacity; ρ is density; T is temperature; λ is thermal conductivity; and Q_t_ is the heat-generation rate. Newton’s law of cooling serves as the foundation for thermal convection on the specimen’s surface during welding, and thermal radiation is based on Stefan–Boltzmann’s law.

(9) $$q_{c}=\alpha_{c}(T-T_{0})$$

(10) $$q_{r}=\varepsilon C_{r}(T-T_{0})$$

where α_c_ is the air heat transfer coefficient, set to be 2 × 10^−5^ W·mm^−2^·°C^−1^; T is the specimen surface temperature; T_0_ is the ambient temperature, set to be 25 °C; C_r_ is the Stefan–Boltzmann coefficient; and ε is the radiation coefficient, set to be 0.8. The thermophysical properties of the metal materials are obtained using JMatPro 7.0, as shown in Figure 10 [29,30].

#### 3.4.2. Mechanical Analysis

First, the transient temperature field is simulated by thermal analysis. Second, the temperature at each time node is loaded into the structural field as thermal, and the residual stresses in the structural field are further calculated by this loading. The same mechanical properties of the WM and the BM are assumed during the mechanical analysis. The high temperature time is too short in the whole-temperature thermal cycling, and low-carbon steel is much less affected by solid-state phase transformation than medium- and high-carbon steels. The small strain rate due to the creep and phase transformation is not considered in this study [31,32]. Excluding the strain rate components due to the creep and phase transformation, the total strain rate is decomposed as:

(11) $$\varepsilon^{tot}=\varepsilon^{e}+\varepsilon^{p}+\varepsilon^{th}$$

where ε^tot^ is total strain; ε^e^ is elastic strain; ε^p^ is plastic strain; and ε^th^ is thermal strain. According to the isotropic Hooke’s law, the temperature-dependent Poisson’s ratio and Young’s modulus are used to simulate the elastic strain. According to the isotropic strain hardening law and the von Mises yield criterion, the yield strength and tensile strength of the material are used to simulate the plastic strain. The yield strength and tensile strength of 410 stainless steel are 477.6 MPa and 573.2 MPa, respectively, and those of RCL540 carbon steel are 513.1 MPa and 588.4 MPa, respectively.

#### 3.4.3. Heat Source Model

**Figure 11:**
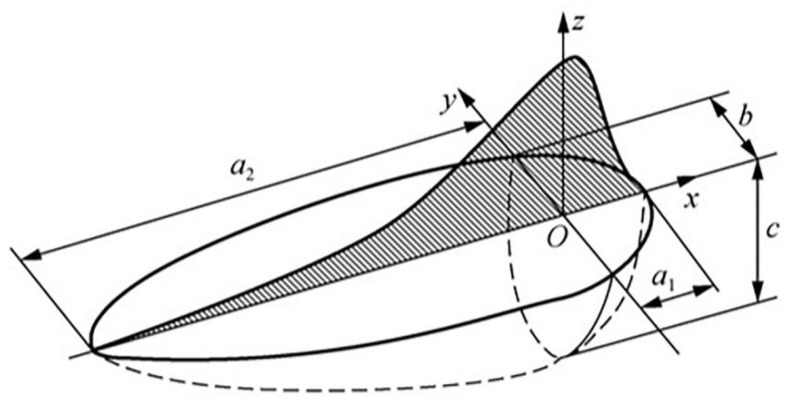
Double-ellipsoidal heat source model.

**Figure 12:**
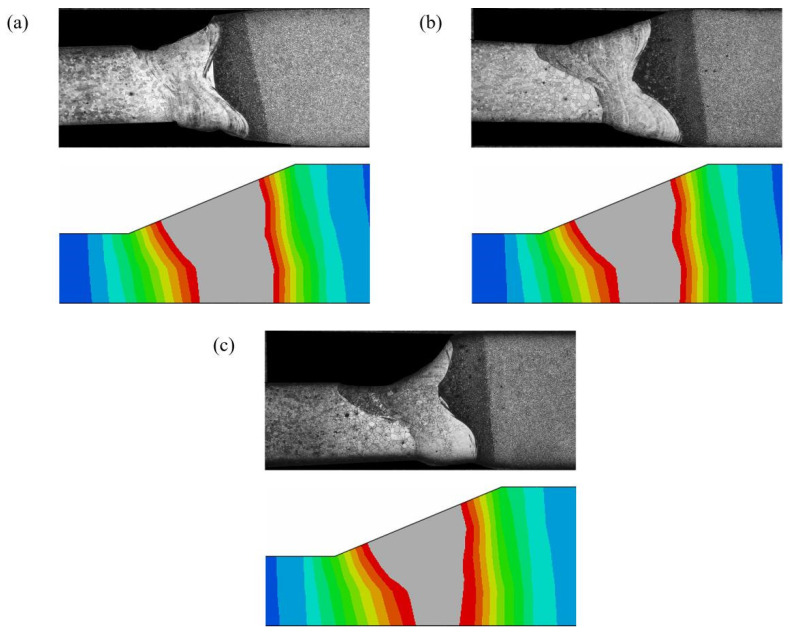
Comparison diagram of weld section morphology. (**a**) 1200 W. (**b**) 1100 W. (**c**) 1000 W.

To simulate the power increase heat flow concentrated in the lower part of the molten pool, we used the DFLUX subroutine (Cheng Jin (2024). AMFlux10) [33] of the combined two double-ellipsoidal heat sources at the same time. The upper part of the heat source is assigned at 600 W, and the lower part of the heat source is assigned at 400 W, 500 W, and 600 W. For the double-ellipsoidal heat source model as shown in Figure 11 [33], the heat source model is divided into front and back parts, and the equations for the heat flow in each part are as follows [34]:

(12) $$q_{1}(x,y,z)=\frac{6\sqrt{3}f_{1}\varphi }{a_{1}bc\pi \sqrt{\pi }}\;exp(-\frac{3x^{2}}{{a_{1}}^{2}}-\frac{3y^{2}}{b^{2}}-\frac{3z^{2}}{c^{2}}),\;x\geq 0$$

(13) $$q_{2}(x,y,z)=\frac{6\sqrt{3}f_{2}\varphi }{a_{2}bc\pi \sqrt{\pi }}\;exp(-\frac{3x^{2}}{{a_{2}}^{2}}-\frac{3y^{2}}{b^{2}}-\frac{3z^{2}}{c^{2}}),\;x<0$$

where q_1_(x, y, z) and q_2_(x, y, z) are the heat flow distributions in the volume of the front and back parts of the ellipsoids; a_1_ is the front part’s axis length of the effective radius of the heat source; a_2_ is the back part’s axis length of the effective radius of the heat source; b is the width of the effective radius of the heat source; c is the depth of the effective radius of the heat source; f_1_ and f_2_ are the energy ratios of the front and back parts of the double-ellipsoidal; and the sum of the two remains constant as follows: f_1_ + f_2_ = 2. Due to the calibration of the parameters of the heat source model, the simulation results of different heat source parameters need to be compared with the optical morphology of the welded joints to determine the appropriate heat source parameters. Figure 12 shows the optical morphology of the weld seam and the simulation cross-section morphology at different powers. The gray part is the area above the melting point of the steel (1500 °C), and calculated results differ very little from theoretical calculations. Finally, the double-ellipsoidal heat source model parameters are obtained after calibration and are as follows: a_1_ = b = 1.5, a_2_ = 3, c = 1, f_1_/f_2_ = 0.5, η_1_ = 1, η_2_ = 0.8, and η_1_ and η_2_ are the thermal efficiencies of the upper and lower parts’ heat sources.

#### 3.4.4. Model Geometry

**Figure 13:**
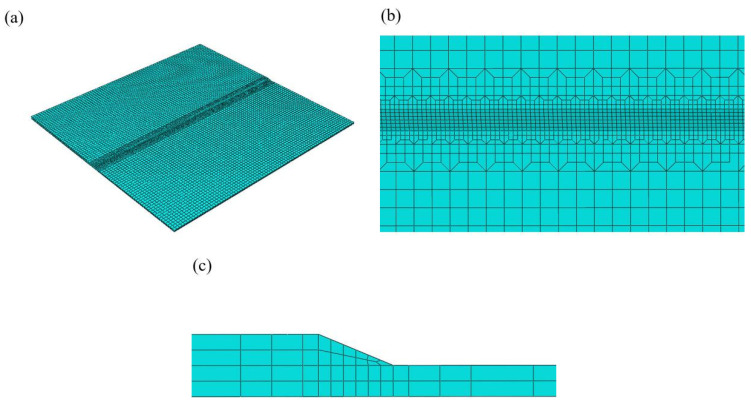
Finite element model of laser welding. (**a**) Overall view of the model. (**b**) Top view of the model. (**c**) Section view of the model.

The actual weld seam shape after welding is used to establish the model to simulate the laser beam incidence angle during welding. ABAQUS 2022 software is used to analyze the different meshes in different parts according to the different ranges of temperature and stress gradient changes. (1) The WM is evenly divided, with a minimum element size of 0.5 mm × 0.5 mm × 0.5 mm. (2) The HAZ is connected to the mesh of the neighboring regions using the grid transition form of 1:2. (3) The BM is roughly divided, with an element size of 2 mm × 2 mm × 0.5 mm. The finite element model of laser welding is shown in Figure 13. The number of nodes are 43,241, and the number of elements are 32,320.

DC3D8 hexahedral linear elements are used for thermal analysis, and C3D8R hexahedral linear elements are used for mechanical analysis. Using the above method for meshing ensures the accuracy of the calculated results in the weld region and also improves the efficiency of the calculation. The weld seam in the additive manufacturing process is simulated using “the element birth-death technique”, where the entire weld seam is divided into 80 analysis steps. Elements of the entire weld are deactivated before the weld begins, representing the elements being “killed”. These elements are constantly reactivated during welding, representing the elements being ”revived” [35].

#### 3.4.5. Results of Thermal Analysis

**Figure 14:**
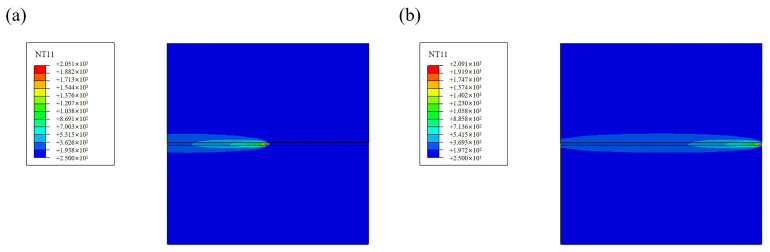

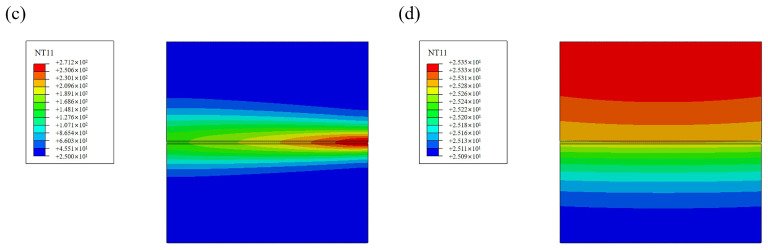
Temperature distribution nephogram at different times. (**a**) t = 3.2 s. (**b**) t = 6.4 s. (**c**) t = 10 s. (**d**) t = 600 s.

The specimen at 1200 W is selected as an example, and instantaneous temperature distribution nephograms of the specimen are analyzed under each moment, shown in Figure 14. From Figure 14a,b, it can be seen that the specimen’s temperature gradually increases during welding. The WM temperature increases to 2051 °C and melts to form the molten pool, which exceeds the melting point of the steels. The molten pool and HAZ move together with the heat source, while the heat source is continuously inputting heat energy. The heat transfer in front of the heat source is less than at the rear, so the maximum temperature of the molten pool will exceed the temperature of the molten pool at steady state. It takes t = 6.4 s to reach the maximum temperature of 2091 °C after the welding heat source no longer inputs heat energy. The temperature of the melt pool drops rapidly to 271 °C when cooling to 10 s, and the cooling rate gradually slows down during the cooling process. When t = 600 s, the temperature drops to the room temperature of 25 °C, and the thermal analysis process is finished.

#### 3.4.6. Results of Mechanical Analysis

**Figure 15:**
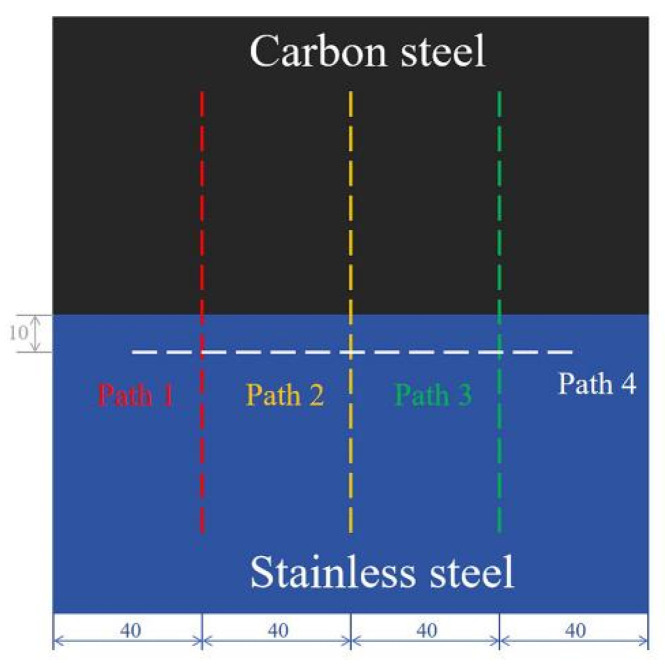
Measurement paths on the top surface.

To investigate the distribution of residual stress in laser welding, four paths are selected in Figure 15.

**Figure 16:**
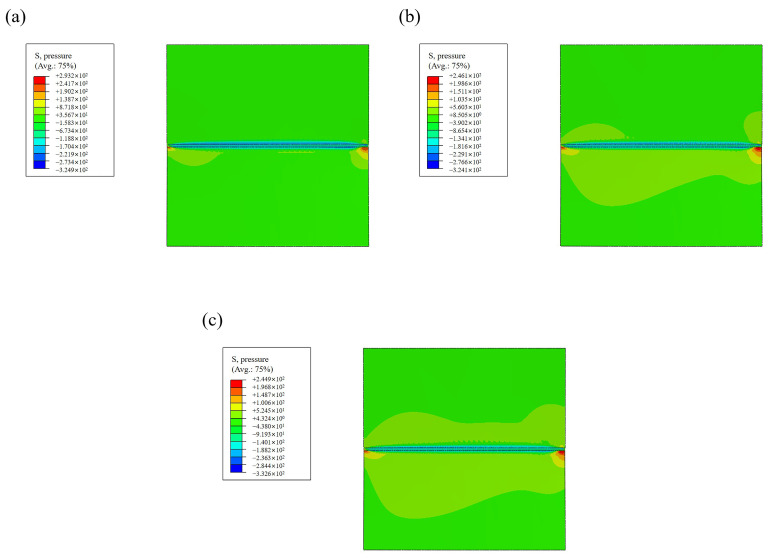
Residual stress distribution nephogram. (**a**) 1200 W. (**b**) 1100 W. (**c**) 1000 W.

**Figure 17:**
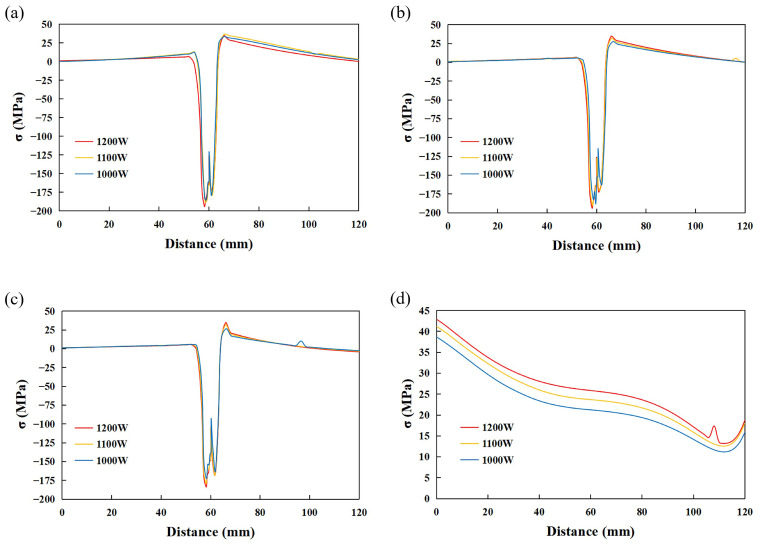
Residual stress value in different paths. (**a**) Path 1. (**b**) Path 2. (**c**) Path 3. (**d**) Path 4.

Path 1, path 2, and path 3 pass through the blind-hole method’s measuring point to measure the longitudinal residual stresses distribution perpendicular to the weld seam. Path 4 passes through the stainless steel side of the measuring point to measure the transverse residual stresses’ distribution parallel to the weld seam. The simulation results of the longitudinal residual stresses’ distribution of the specimen are shown in Figure 16, and the residual stresses’ distribution of different paths are shown in Figure 17. A comparison of Figure 16 with Figure 17a–c shows that the compressive stresses in the WM and the tensile stresses in the WM are the greatest at 1200 W. In Figure 17d, not only do the tensile stresses decrease with decreasing laser power, but also the transverse residual stresses decrease along the welding direction.

**Figure 18:**
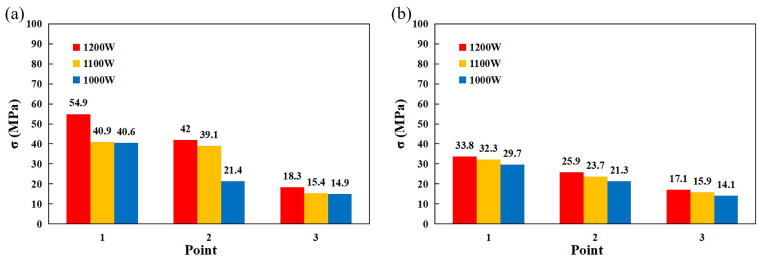
(**a**) Difference in compressive stress measured by blind-hole method. (**b**) Tensile stresses simulated by FEA.

Figure 18a shows the numerical difference in transverse compressive stresses in the stainless steel side measured by the blind-hole method. Figure 18b shows the simulated tensile stresses at the same location with a slight error due to the unevenness of the specimen when measured by the blind-hole method [36]. A comparison of the results shows that the simulation results are consistent with the actual welding residual stress distribution, which verifies the reliability of the FEA.

## 4. Conclusions

In this study, laser welding of sheets of unequal thickness of 410 stainless steel and RCL540 carbon steel are accomplished, and the laser welded joint of 410 ferritic stainless steel and RCL540 low-carbon alloy steel and dissimilar steels are analyzed and investigated. The main conclusions are as follows:

(1) With the increase of laser power, the upper and lower melting width ratios decrease. Due to a keyhole deeper into the specimen, the specimen has a greater absorption of the laser, and the energy is concentrated mainly in the lower part of the molten pool. The welded joint has the best weld formability at 1200 W.
(2) The average Vicker’s hardness of the WM is greater than the stainless steel and carbon steel BM. All specimens fractured in the weak zone of the base metal on the stainless steel side at 10 mm from the weld metal, and laser power has a small effect on yield strength and maximum test force.
(3) Residual stresses are mainly concentrated on the stainless steel side with good uniformity. The stainless steel side is mainly affected by compressive stresses, which decrease as the power increases. The carbon steel side is mainly affected by tensile stresses, which increase with the increase of power.
(4) The tensile stress in the BM simulated by FEA is greatest at 1200 W. As the power decreases along the welding direction, the tensile stress decreases. The results showed that the stainless steel side produces tensile stresses that increase with increasing power offset by compressive stresses in the BM.

## Figures and Tables

**Table 1:** Composition of 410 stainless steel and RCL540 carbon steel (mass fraction Wt %).

Brand	C	Si	Mn	P	S	Cr	Ni	Other
410 stainless steel	0.08	1.00	1.00	0.045	0.03	11.5	0.50	0.75
RCL540 carbon steel	0.061	0.038	1.094	0.021	0.004	0	0	0.029

**Table 2:** Melting width measurement results, heat input, and power density calculation results.

Laser Power P/W	Heat Input HI/J·mm^−1^	Power Density ρ/W·cm^−2^	Upper Melting Width d_1_/mm	Lower Melting Width d_2_/mm
1200	48	1.70×10^6^	1.31	1.12
1100	44	1.56×10^6^	1.64	1.15
1000	40	1.42×10^6^	1.56	0.86

## Data Availability

The datasets used and analyzed during the current study are available from the corresponding author on reasonable request.
